# A preliminary result of three-dimensional microarray technology to gene analysis with endoscopic ultrasound-guided fine-needle aspiration specimens and pancreatic juices

**DOI:** 10.1186/1756-9966-29-36

**Published:** 2010-04-25

**Authors:** Koji Nonogaki, Akihiro Itoh, Hiroki Kawashima, Eizaburo Ohno, Takuya Ishikawa, Hiroshi Matsubara, Yuya Itoh, Yosuke Nakamura, Masanao Nakamura, Ryoji Miyahara, Naoki Ohmiya, Masatoshi Ishigami, Yoshiaki Katano, Hidemi Goto, Yoshiki Hirooka

**Affiliations:** 1 Department of Gastroenterology, Nagoya University Graduate School of Medicine, 65 Tsuruma-cho, Showa-ku, Nagoya 4668550, Japan; 2 Department of Endosocpy, Nagoya University Hospital, 65 Tsuruma-cho, Showa-ku, Nagoya 4668550, Japan

## Abstract

**Background:**

Analysis of gene expression and gene mutation may add information to be different from ordinary pathological tissue diagnosis. Since samples obtained endoscopically are very small, it is desired that more sensitive technology is developed for gene analysis. We investigated whether gene expression and gene mutation analysis by newly developed ultra-sensitive three-dimensional (3D) microarray is possible using small amount samples from endoscopic ultrasound-guided fine-needle aspiration (EUS-FNA) specimens and pancreatic juices.

**Methods:**

Small amount samples from 17 EUS-FNA specimens and 16 pancreatic juices were obtained. After nucleic acid extraction, the samples were amplified with labeling and analyzed by the 3D microarray.

**Results:**

The analyzable rate with the microarray was 46% (6/13) in EUS-FNA specimens of RNAlater^® ^storage, and RNA degradations were observed in all the samples of frozen storage. In pancreatic juices, the analyzable rate was 67% (4/6) in frozen storage samples and 20% (2/10) in RNAlater^® ^storage. EUS-FNA specimens were classified into cancer and non-cancer by gene expression analysis and K-ras codon 12 mutations were also detected using the 3D microarray.

**Conclusions:**

Gene analysis from small amount samples obtained endoscopically was possible by newly developed 3D microarray technology. High quality RNA from EUS-FNA samples were obtained and remained in good condition only using RNA stabilizer. In contrast, high quality RNA from pancreatic juice samples were obtained only in frozen storage without RNA stabilizer.

## Background

**Figure 1:**
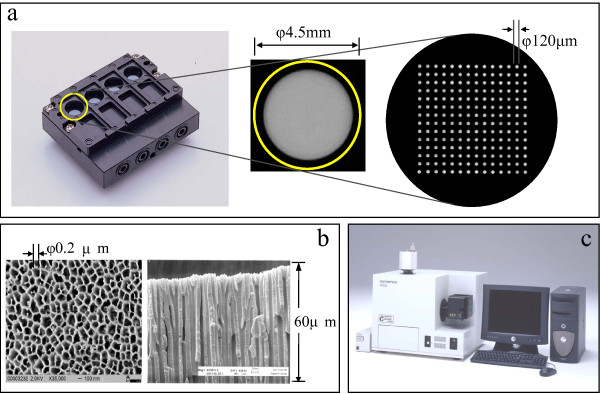
**Schematic description of newly developed 3D microarray technology**. (a) The 3D microarray (PamChip) with the four array format (left), an array with a diameter of 45 mm (middle), and a set of oligo DNA probes immobilized with 120 μm diameter (right). (b) The partial top (left) and cross section view (right) of multi-porous substrate within PamChip. (c) FD10 microarray system with functions of hybridization, washing, fluorescence imaging and image analysis, which are integrated and performed semi-automatically.

**Figure 2:**
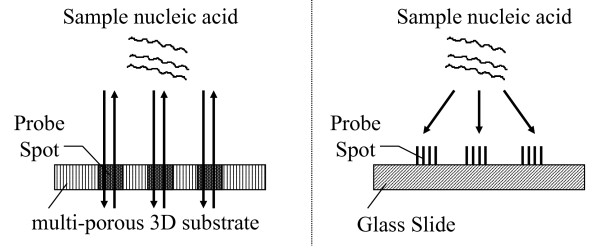
**Comparison between 3D microarray (left) and conventional 2D microarray (right)**.

The microarray is the advanced technology that is useful for comprehensive gene expression profiling. Microarray analysis has the possibility of identifying subsets of genes that may be especially useful markers for cancer diagnosis [1,2]. The Olympus Co.Ltd. (Tokyo, Japan) has developed a novel microarray technology, the three dimensional (3D) microarray system in cooperation with PamGene International, B.V. (BJ's-Hertogenbosch, The Netherlands). This technology involves the use of a multi-porous 3D substrate and flow-through hybridization technique with pumping sample solution rapidly and semi-automatically (Figure 1). Unlike conventional microarrays that use a 2D substrate such as a slide glass, the 3D microarray binds a huge amount of probe DNA molecules onto a solid 3D structure substrate (Figure 2) as previously described [3,4].

Recently, detailed, global, genomic analyses have lead to a better understanding of the pathogenesis of pancreatic tumors. This has opened up avenues for the development of novel diagnostic and individually tailored treatment strategies [5-9]. Microarrays have traditionally been applied to pancreatic tissue obtained from surgical resection, but in this report, we investigated whether gene analysis by 3D microarray is possible using small samples obtained endoscopically for the pancreatic lesions.

## Methods

### Samples

This study was approved by the Institutional Review Board of Nagoya University Graduate School of Medicine. Written informed consents were obtained from all patients. Seventeen endoscopic ultrasound-guided fine-needle aspiration (EUS-FNA) specimens, pancreatic adenocarcinoma (n = 11), chronic pancreatitis (n = 3), autoimmune pancreatitis (n = 2) and pancreatic endocrine tumor (n = 1), and 16 pancreatic juices, pancreatic adenocarcinoma (n = 1), chronic pancreatitis (n = 10) and intraductal papillary mucinous neoplasms (n = 5) were obtained in Nagoya University hospital. EUS-FNA was carried out and the obtained samples were immediately frozen in liquid nitrogen and stored at -80°C or immersed in RNAlater^® ^(Ambion Inc., Austin TX, USA) at 4°C for 16 hours and then stored at -20°C. Pancreatic juices samples were obtained by endoscopic retrograde cholangiopancreatography (ERCP) and immediately frozen in liquid nitrogen and stored at -80°C or mixed with 10 volume of RNAlater^® ^at 4°C for 16 hours and then stored at -20°C. The endoscope and needles used for EUS-FNA was GF-UCT 240 and NA-200H-8022 (22 gauge) (Olympus Co. Ltd. Tokyo Japan). The endoscope and catheters used for ERCP was JF-260V and PR-109-Q-1 (Olympus Co. Ltd. Tokyo Japan).

### Total RNA/DNA extraction

Both total RNA and genomic DNA were simultaneously extracted from the same sample by ISOGEN (NIPPON GENE Inc., Tokyo, Japan). EUS-FNA specimens were pounded in a mortar with liquid nitrogen before extraction of the nucleic acids. Pancreatic juices stored by freezing at -80°C were diluted with 10 volumes of PBS and centrifuged by 2000 rpm for 10 minutes. The obtained pellets were used for nucleic acid extraction. Pancreatic juices stored by RNAlater^® ^were centrifuged by 2000 rpm for 10 minutes. The obtained pellets were used for nucleic acid extraction. The concentration of the obtained nucleic acids was estimated by measuring the optical density (OD) at 260 nm using a Nanodrop (Nanodrop Inc., Wilmington, DE, USA) and their quality was checked by electrophoresis using a Bioanalyzer (Agilent Inc., Santa Clara, CA, USA).

### Gene expression analysis

The 0.1-2 μg of total RNA derived from each sample was amplified as aRNA by Eberwine's method using a Message Amp™ aRNA kit (Ambion Inc.) and labeled with biotin-16-UTP (Roche Inc.) [10].

Hybridization and image analysis were performed using a 3D microarray (PamChip) and FD10 microarray system developed by the Olympus Corporation. The microarray was set up with 60 mer oligo DNA probes of 60 genes: human gene related cancer, pancreatic enzyme, β-actin (ACTB) and glyceraldehyde-3-phosphate dehydrogenase (GAPDH) as house keeping genes and lambda DNA (LAMD) and renilla luciferase gene (pRL-TK) as negative controls. Each probe sequence was designed by Novusgene Inc. Hybridization, washing and fluorescence detection were performed semi-automatically in the FD10. The 50 ng of each labeled aRNA was dissolved in 3XSSPE, including 0.5% Lauryl sarcosine and applied on Pamchip and hybridization was performed at 42°C for 1.5 hours. After the hybridization reaction, the Pamchip was washed and fluorescent signals were amplified using an enzymatic reaction kit (TSA™ Kit #22, Invitogen Inc., Carlsbad, CA, USA). The CCD images were automatically taken by the FD10 and each image was analyzed by the original analysis software. Hierarchical clustering by UPGMA methods and the Welch *t *statistic were performed using Spotfire Decision Site Functional Genomics ver.8.0 (Spotfire Inc., PaloAlto, CA, USA).

### Gene mutation analysis (K-ras codon 12/13)

The 50 ng of genomic DNA were amplified by Ex-taq polymerase (TaKaRa, Kyoto, Japan) and labeled by PCR with fluorescent (FITC) labeled primers. PCR was performed under conditions of 94°C:1 min, 55°C:2 min, 72°C:1 min. (35 cycles). The forward and the reverse primer sequence is GACTGAATATAAACTTGTGG and CTATTGTTGGATCATATTCG, respectively. Hybridization and Image analysis were performed using FD10, according to the procedure by Maekawa et al [11].

## Results

### Sample preparation

Both total RNA and genomic DNA were extracted from each EUS-FNA specimen (See Table S1, Additional file 1) and pancreatic juice (See Table S2, Additional file 2). In EUS-FNA specimens, the weight of each specimen was in the range from 10 to 200 mg. The average amounts of obtained total RNA were 4.92 ± 3.09 μg (n = 4) (260/280:1.68 ± 0.26) at frozen storage and 2.51 ± 3.49 μg (n = 13) (260/280:1.70 ± 0.14) at RNAlater^® ^storage, respectively. In each of the frozen samples of pancreatic juices, pellets were formed in gel-like form. On the other hand, in each of the RNA later-storage samples of pancreatic juices, white pellet were formed. The average amounts of obtained total RNA were 3.94 ± 3.98 μg (n = 6) (260/280:1.63 ± 0.23) at frozen storage and 0.44 ± 0.66 μg (n = 10) (260/280:1.55 ± 0.31) at RNAlater^® ^storage, respectively. Only small total RNA could be obtained by samples of RNAlater^® ^storage.

The quality and degradation of total RNA was checked by electrophoresis. In EUS-FNA specimens, RNA degradations were observed in all the samples of frozen storage. On the other hand, in RNAlater^® ^stored samples, 5 of 13 samples showed both bands of 16 s and 28 s rRNA. In pancreatic juice samples, almost all sample of frozen storage showed two bands of rRNA, but in RNAlater^® ^stored samples, almost all samples showed RNA degradations. After the treatment with DNase, the 0.1-2 μg of total RNA was amplified using Eberwine's method. The average of aRNA amplifications in EUS-FNA specimens were 129 ± 99 and 252 ± 253 fold in frozen and RNAlater^® ^storage, respectively. In pancreatic juices samples, 298 ± 142 and 235 ± 149 in frozen and RNAlater^® ^storage, respectively. The RNA sample with good quality confirmed by electrophoresis showed efficient aRNA amplification (Table S1, Additional file 1 and Table S2, Additional file 2).

### Gene Expression Analysis

We optimized the technique of enzymatic hybridization signal amplification by applying TSA technology to the 3D structure of our microarray [12]. As a result, fluorescent molecules accumulated at the surface of the multiple pores, and approximately 1000-fold signal amplification was realized when compared with the conventional microarray method. Each hybridization was performed with only 50 ng of aRNA labeled with biotin.

The samples with two-bands of rRNAs in electrophoresis and with an efficient rate of aRNA amplification (over 300-fold) were analyzable on the microarray hybridization showing sufficient signal intensity on most of the spots. However, the other samples did not hybridize on the microarray at all. The analyzable rate with the microarray was 46% (6/13) in EUS-FNA specimens of RNAlater^® ^storage. In pancreatic juices, analyzable rate was 67% (4/6) in frozen storage samples and 20% (2/10) in RNAlater^® ^storage. After each hybridization, hybridization images were automatically taken by the CCD camera integrated in the FD10, and original image analysis software calculated the fluorescence intensity of each spot and subtracted the background value. Six of those data from EUS-FNA specimens and six data from the pancreatic juice previously obtained were applied to hierarchical clustering analysis using Spotfire DecisionSite Functional Genomics http://www.spotfire.com/ with 25 genes, which showed sufficient signal intensity in most of the samples.

**Figure 3:**
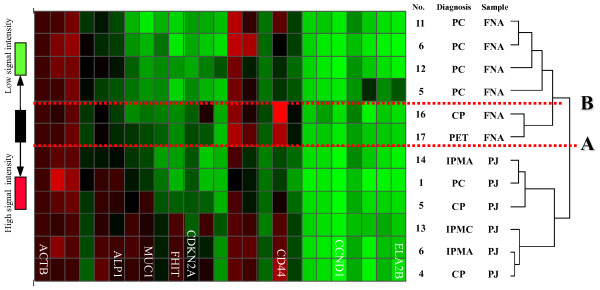
**Hierarchical cluster of the human 25 genes expression pattern in 12 pancreatic samples**. FNA, EUS-FNA specimens (n = 6); PJ, pancreatic juice samples (n = 6); PC, pancreatic cancer (n = 5); CP, chronic pancreatitis (n = 3); IPMC, intraductal papillary mucinous adenocarcinoma (n = 1); IPMA, intraductal papillary mucinous adenoma (n = 2); PET, pancreatic endocrine tumor (n = 1). Each color scale represents the signal intensity of each gene. Some genes that significantly contributed to the dividing of clusters (p < 0.005) were noted at the bottom of the panel. Line A shows the boundary of the gene expression pattern between EUS-FNA and pancreatic juice. Line B shows the boundary of cancer or non-cancer in the EUS-FNA specimens.

In the gene expression analysis, the samples were classified into two clusters, EUS-FNA samples and pancreatic juice samples (pellets after centrifugation), by the 1^st ^clustering (Figure 3, line A). The cluster of the EUS-FNA sample was further classified into cancerous or non-cancerous clusters by the 2^nd ^clustering (Figure 3, line B). Conversely, the cluster of pancreatic juice samples were not clearly classified as cancerous and non-cancerous cluster. The genes, ALP1, MUC1, CCND1, CDK2 and FHIT significantly contributed to the 1^st ^clustering between the EUS-FNA samples and the pancreatic juice samples (p < 0.05). On the other hand, in the EUS-FNA samples, the gene, CDK2A, CD44, S100A4 and MUC1 were specifically related to the 2^nd ^clustering between cancer and non-cancer (p < 0.05).

### Gene mutation analysis (K-ras codon 12/13)

PCR amplification and gene mutation analysis for K-ras (codon12/13) were successful in the case of the samples with good quality total RNA. We extracted the total RNA and DNA from the same specimens in this study. When one nucleic acid could not be successfully prepared and analyzed, the other nucleic acid also could not be used. The degradation of the nucleic acid seems to be depended on the condition of sample storage after EUS-FNA or collecting pancreatic juices. All of the analyzable pancreatic cancer samples showed a specific mutation of K-ras codon12 with a single base change from GGT (Gly) to GAT (Asp) (See Table S3, Additional file 3), which is the most frequent mutation, as previously reported [13]. Additionally, one of the analyzable chronic pancreatitis samples showed a mutation from GGT (Gly) to GTT (Val), which is also frequent in pancreatic cancer as previously reported. No mutation could be detected in the samples of autoimmune pancreatitis and pancreatic endocrine tumor.

## Disscussion

DNA microarrays can analyze plural gene expression changes at the same time. It is useful for the early detection of pancreatic cancer, evaluation of malignant potential and drug efficacy. There are some articles about the identification of genes that show chemosensitivity to anti-cancer drugs, such as gemcitabine and 5FU in the pancreatic cancer cell line [14,15]. Gene expression profiling would especially help to predict the effectiveness of chemotherapy. This time, we inspected whether gene expression analysis by 3D microarray was possible using small amount samples obtained endoscopically.

In EUS-FNA specimens, the sample storage method using RNAlater^® ^seemed to improve the quality of the total RNA when compared with the method using liquid nitrogen storage. Since pancreatic tissues have large amounts of digestive enzymes, i.e., protease and/or nuclease, nucleic acids could easily be degraded. Furthermore, since EUS-FNA specimens are long and thin, they may be easily broken down by digestive enzymes. On the other hand, a reagent such as an RNase inhibitor included in RNAlater^® ^may be easy to instill into the tissues and/or their cell components for the same reason. Therefore, EUS-FNA specimens may be suitable for storage with RNAlater^® ^for RNA preparation. In our investigation, the analyzable rate was lower than 50% for EUS-FNA specimens of RNAlater^® ^storage (46%). For further improvement, it will be very important to take as many cell-rich EUS-FNA specimens as possible. Actually, specimens that we couldn't obtain from contained much fibrotic tissue or blood instead of cells (data not shown). After EUS-FNA, confirmation of the cell component by microscopic observation and preservation of only cell-rich part with RNAlater^® ^cutting off from the obtained specimens will be efficient before RNA preparation.

In pancreatic juice samples, total RNA and DNA were obtained in good quality and quantity from the directly frozen samples. RNAlater^® ^storage could not improve quality of nucleic acid in pancreatic juice. All those samples involved white pellet. We suspected that the component of white pellet was a contrast agent contained in the pancreatic juice samples. To confirm it, we mixed RNAlater^® ^and the contrast agent Urografin^®^, and the white pellets like in RNAlater^®^-stored samples were observed immediately. Furthermore, the volume of the white pellets appeared were almost the same as that of Urografin^®^. The contrast agent is difficult to be dissolved, therefore, when it is mixed with different solution such as RNAlater^®^, its composition changes and the contrast agent may precipitate. If we use RNAlater^® ^for pancreatic juice storage, we have to remove the supernatant containing a contrast agent such as Urografin^®^, for example, by performing centrifuge. After then, only the precipitation including pancreatic cells should be stored with RNAlater^®^. Furthermore, control experiments with RNase inhibitors other than RNAlater^® ^to exclude the possible vehicle effects will be needed. Pancreatic juice is an ideal specimen for pancreatic cancer biomarkers discovery, because it is an exceptionally rich source of proteins released from pancreatic cancer cells [16-18]. Gene analysis of pancreatic juice deserves further investigation to determine its utility as a tool for the evaluation of pancreatic lesions.

It may be presumed that FNA samples and pancreatic juice samples were classified into different clusters because the cell population is different in the two kinds of samples. The gene expression data obtained in this study succeeded in classifying cancer and non-cancer in the EUS-FNA samples. However, the pancreatic juice samples were not classified as any particular cluster. One of the reasons may be that the pancreatic juice samples were composed of various kinds of diagnostic samples.

Patient-tailored medicine can be defined as the selection of specific therapeutics to treat disease in a particular individual based on genetic, genomic or proteomic information. While individualized treatments have been used in medicine for many years, advances in cancer treatment have now generated a need to more precisely define and identify those patients who will derive the most benefit from new-targeted agents [19,20]. We succeeded in gene expression analysis and gene mutation analysis using the small amount samples by the newly developed 3D microarray system. The gene expression analysis and gene mutation analysis requires only 2 days and 4 hours after the isolation of samples to obtain data. The 3D microarray has potential for providing detailed information about the pancreatic lesions from small samples such as EUS-FNA specimens and pancreatic juices.

It is very difficult to correctly determine the detection limit of microarray analysis for mRNA expression pattern and mutation identification. However, from the viewpoint of clinical use, we recommend that at least 0.1-2 ug of total RNA will be sufficient for mRNA expression analysis. On the other hand, for gene mutation analysis, 50 ng of genomic DNA were used for conventional PCR in this study. The detection limit of mutant alleles by the 3D microarray is estimated to be 16-25% of the total genomic DNA as previously reported [11].

## Conclusions

Gene analysis from small amount samples obtained endoscopically was possible by newly developed 3D microarray technology. High quality RNA from EUS-FNA samples were obtained and remained in good condition only using RNA stabilizer. In contrast, high quality RNA from pancreatic juice samples were obtained only in frozen storage without RNA stabilizer.

## Abbreviations

3D microarray: three-dimensional microarray; EUS-FNA: endoscopic ultrasound-guided fine-needle aspiration; ERCP: endoscopic retrograde cholangiopancreatography;

## Competing interests

The authors declare that they have no competing interests.

## Authors' contributions

KN, AI, HG and YH made conception, designed and coordinated the study, collected samples, analyzed data, carried out data interpretation, and drafted the manuscript. HK, EO, TI, HM, YI, and YN collected samples and evaluated the results. MN, RM, NO, MI and YK participated in the conception, analyzed data, carried out data interpretation, design of study and in drafting of manuscript. All authors read and approved the final manuscript

## Supplementary Material

Additional file 1 **Table S1: Summary of each EUS-FNA specimen and obtained RNA/DNA information**. In EUS-FNA specimens, RNA degradations were observed in all the samples of frozen storage. On the other hand, in RNAlater^® ^stored samples, 5 of 13 samples were in good conditions. Click here for file

Additional file 2 **Table S2: Summary of each pancreatic juice sample and obtained RNA/DNA information**. In pancreatic juice samples, almost all sample of frozen storage were in good conditions, but in RNAlater^® ^stored samples, almost all samples showed RNA degradations. Click here for file

Additional file 3 **Table S3: Result of gene mutation analysis of K-ras codon 12/13 (left: EUS-FNA specimens, right: Pancreatic juices)**. All of the analyzable pancreatic cancer samples showed a specific mutation of K-ras codon12 with a single base change from GGT (Gly) to GAT (Asp). Click here for file
