# Supplementary material for: A preliminary result of three-dimensional microarray technology to gene analysis with endoscopic ultrasound-guided fine-needle aspiration specimens and pancreatic juices
Source: J Exp Clin Cancer Res. 2010 Apr 25;29(1):36. doi: 10.1186/1756-9966-29-36 (PMC2867810; doi:10.1186/1756-9966-29-36)
Supplement: Additional file 1 — Table S1: Summary of each EUS-FNA specimen and obtained RNA/DNA information. In EUS-FNA specimens, RNA degradations were observed in all the samples of frozen storage. On the other hand, in RNAlater® stored samples, 5 of 13 samples were in good conditions. [file 1756-9966-29-36-S1.DOC]

| Sample  No. | Diagnosis | Sample storage | Sample volume  (mg) | Appearance | RNA | | | aRNA amplification | | | | DNA | |
| --- | --- | --- | --- | --- | --- | --- | --- | --- | --- | --- | --- | --- | --- |
|  |  |  |  |  | totalRNA  (μg) | OD ratio  (260/280) | Electro-  phoresis | applied  totalRNA  (μg） | obtained  aRNA  (μg） | fold  of aRNA amplificaion | hybridi-  zation | DNA  (μg) | OD ratio  (260/280) |
| 1 | PC | liquid N2 frozen | 200 | string-like | 7.7 | 2.05 | × | 2.0 | 4.0 | 100 | × | ND | ND |
| 2 | PC | liquid N2 frozen | 100> | string-like | 1.4 | 1.57 | × | 0.3 | 0.5 | 107 | × | ND | ND |
| 3 | PC | liquid N2 frozen | 200 | string-like | 7.3 | 1.62 | × | 2.0 | 10.5 | 263 | × | 4.0 | 1.76 |
| 4 | AP | liquid N2 frozen | 200 | string-like | 3.4 | 1.47 | × | 2.0 | 1.1 | 28 | × | 1.8 | 1.35 |
| 5 | PC | RNAlater | 200 | string-like | 8.0 | 1.75 | ○ | 1.0 | 13.5 | 767 | ○ | 1.6 | 1.20 |
| 6 | PC | RNAlater | 100> | string-like | 2.2 | 1.83 | ○ | 0.5 | 2.7 | 269 | ○ | 0.5 | 1.10 |
| 7 | PC | RNAlater | 100> | string-like | 1.0 | 1.88 | × | 0.2 | 0.2 | 75 | × | ND | ND |
| 8 | PC | RNAlater | 100> | string-like | 0.5 | 1.64 | × | 0.2 | 0.2 | 102 | × | ND | ND |
| 9 | PC | RNAlater | 70 | string-like | 1.1 | 1.80 | × | 0.5 | 0.7 | 70 | × | ND | ND |
| 10 | PC | RNAlater | 260 | string-like | 0.6 | 1.77 | × | 0.5 | 0.0 | 0 | × | ND | ND |
| 11 | PC | RNAlater | 100> | fragment | 0.7 | 1.92 | ○ | 0.6 | 6.9 | 541 | ○ | 0.7 | 1.56 |
| 12 | PC | RNAlater | 50> | string-like | 12.0 | 1.78 | ○ | 0.6 | 6.0 | 507 | ○ | 0.6 | 1.34 |
| 13 | AP | RNAlater | 130 | string-like | 0.7 | 1.60 | × | 0.5 | 0.3 | 30 | × | ND | ND |
| 14 | CP | RNAlater | 100 | string-like | 1.4 | 1.48 | × | 0.3 | 0.4 | 74 | × | 0.3 | 1.32 |
| 15 | CP | RNAlater | 10> | fragment | 1.5 | 1.43 | × | 0.5 | 0.0 | 0 | × | 0.3 | 1.35 |
| 16 | CP | RNAlater | 50> | fragment | 0.2 | 1.76 | ○ | 0.5 | 3.5 | 367 | ○ | 0.5 | 1.26 |
| 17 | PET | RNAlater | 100> | fragment | 2.80 | 1.65 | ○ | 1.0 | 9.4 | 469 | ○ | 2.8 | 1.65 |

Table S1. Summary of each EUS-FNA specimen and obtained RNA/DNA information

PC, pancreatic cancer; CP, chronic pancreatitis; AP, autoimmune pancreatitis; PET, pancreatic endocrine tumor;

ND, not-detected.
