# Supplementary material for: A preliminary result of three-dimensional microarray technology to gene analysis with endoscopic ultrasound-guided fine-needle aspiration specimens and pancreatic juices
Source: J Exp Clin Cancer Res. 2010 Apr 25;29(1):36. doi: 10.1186/1756-9966-29-36 (PMC2867810; doi:10.1186/1756-9966-29-36)
Supplement: Additional file 2 — Table S2: Summary of each pancreatic juice sample and obtained RNA/DNA information. In pancreatic juice samples, almost all sample of frozen storage were in good conditions, but in RNAlater® stored samples, almost all samples showed RNA degradations. [file 1756-9966-29-36-S2.PPT]

## Slide 1
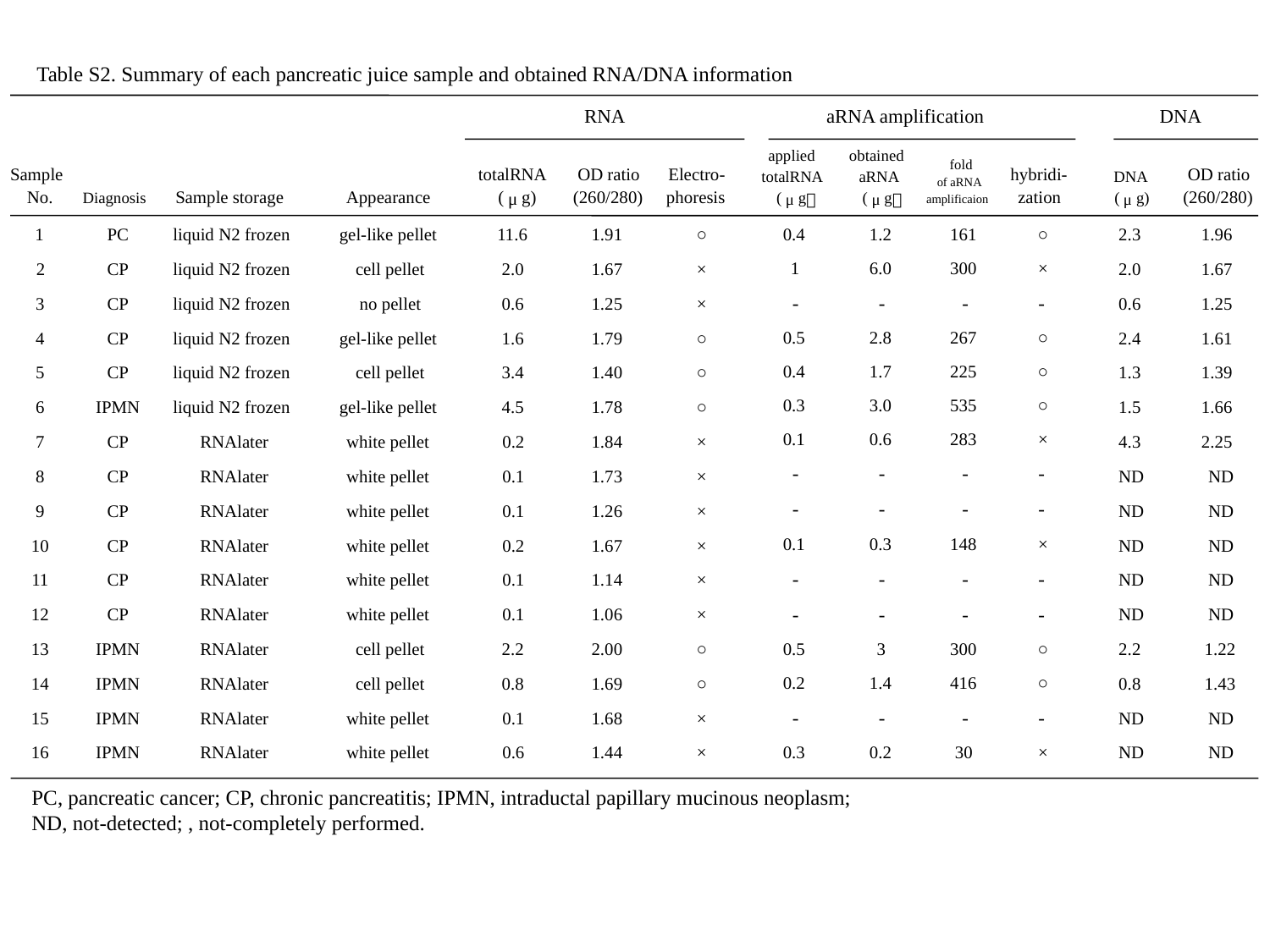

Table S2. Summary of each pancreatic juice sample and obtained RNA/DNA information
RNA
aRNA amplification
DNA
applied
obtained
fold
Sample
totalRNA
 OD ratio
Electro-
hybridi-
 OD ratio
 totalRNA
aRNA
DNA
of aRNA
No.
Sample storage
Appearance
(
g)
(260/280)
phoresis
zation
(260/280)
Diagnosis
(
g
(
g
(
g)
μ
μ
）
μ
）
μ
amplificaion
1
PC
liquid N2 frozen
gel-like pellet
11.6
1.91
○
0.4
1.2
161
○
2.3
1.96
1
6.0
300
×
2
CP
liquid N2 frozen
cell pellet
2.0
1.67
×
2.0
1.67
-
-
-
-
 3
CP
liquid N2 frozen
no pellet
0.6
1.25
×
0.6
1.25
0.5
2.8
267
○
 4
CP
liquid N2 frozen
gel-like pellet
1.6
1.79
○
2.4
1.61
0.4
1.7
225
○
 5
CP
liquid N2 frozen
cell pellet
3.4
1.40
○
1.3
1.39
0.3
3.0
535
○
 6
IPMN
liquid N2 frozen
gel-like pellet
4.5
1.78
○
1.5
1.66
0.1
0.6
283
×
 7
CP
RNAlater
white pellet
0.2
1.84
×
4.3
2.25
-
-
-
-
 8
CP
RNAlater
white pellet
0.1
1.73
×
ND
ND
-
-
-
-
 9
CP
RNAlater
white pellet
0.1
1.26
×
ND
ND
0.1
0.3
148
×
10
CP
RNAlater
white pellet
0.2
1.67
×
ND
ND
-
-
-
-
11
CP
RNAlater
white pellet
0.1
1.14
×
ND
ND
-
-
-
-
12
CP
RNAlater
white pellet
0.1
1.06
×
ND
ND
13
IPMN
RNAlater
cell pellet
2.2
2.00
○
2.2
1.22
0.5
3
300
○
0.2
1.4
416
○
14
IPMN
RNAlater
cell pellet
0.8
1.69
○
0.8
1.43
-
-
-
-
15
IPMN
RNAlater
white pellet
0.1
1.68
×
ND
ND
16
IPMN
RNAlater
white pellet
0.6
1.44
×
0.3
0.2
30
×
ND
ND
PC, pancreatic cancer; CP, chronic pancreatitis; IPMN, intraductal papillary mucinous neoplasm;
ND, not-detected; , not-completely performed.
