# Supplementary material for: A preliminary result of three-dimensional microarray technology to gene analysis with endoscopic ultrasound-guided fine-needle aspiration specimens and pancreatic juices
Source: J Exp Clin Cancer Res. 2010 Apr 25;29(1):36. doi: 10.1186/1756-9966-29-36 (PMC2867810; doi:10.1186/1756-9966-29-36)
Supplement: Additional file 3 — Table S3: Result of gene mutation analysis of K-ras codon 12/13 (left: EUS-FNA specimens, right: Pancreatic juices). All of the analyzable pancreatic cancer samples showed a specific mutation of K-ras codon12 with a single base change from GGT (Gly) to GAT (Asp). [file 1756-9966-29-36-S3.PPT]

## Slide 1
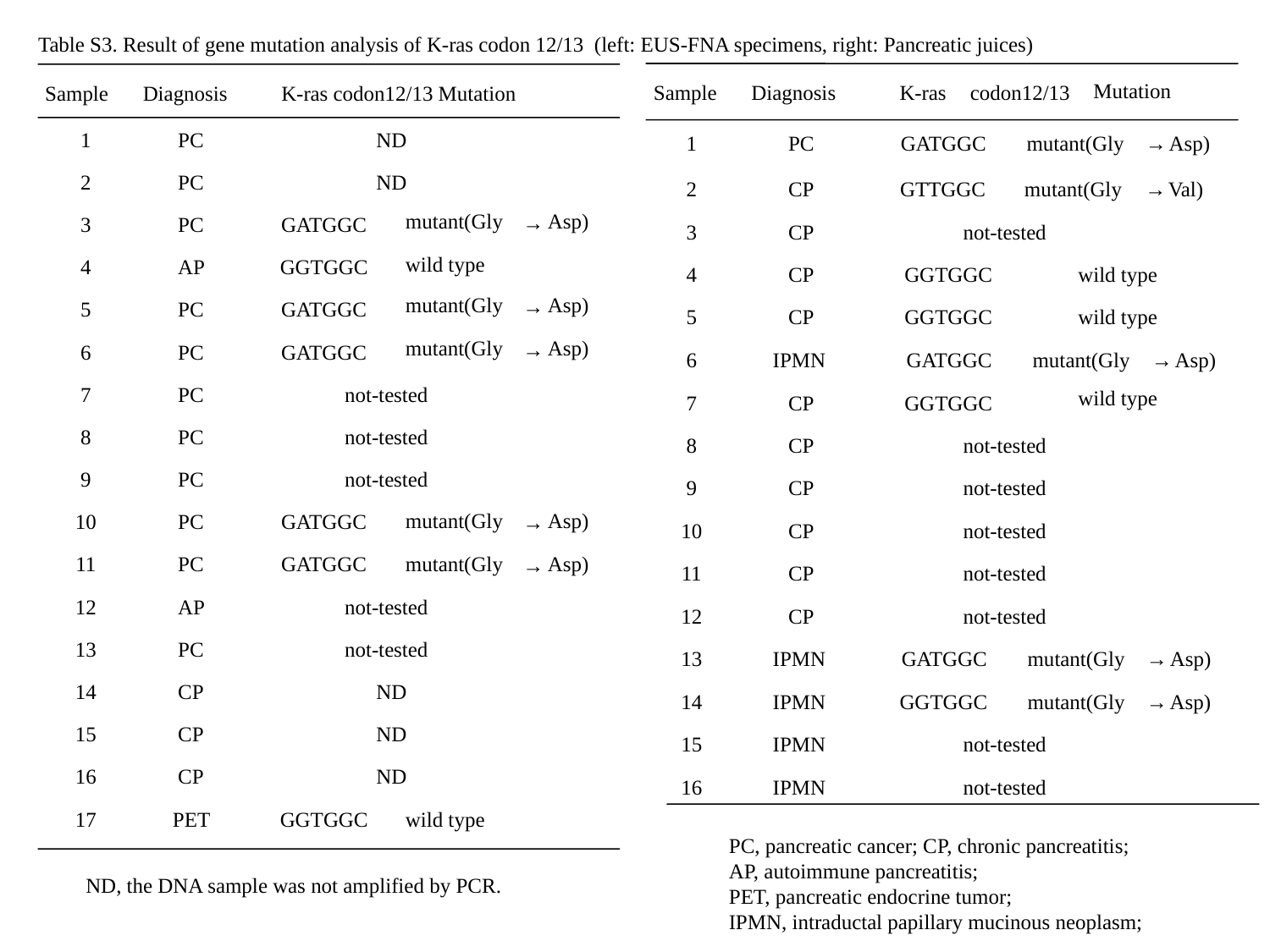

Table S3. Result of gene mutation analysis of K-ras codon 12/13 (left: EUS-FNA specimens, right: Pancreatic juices)
Mutation
Sample
Diagnosis
K-ras
codon12/13
K-ras codon12/13 Mutation
Sample
Diagnosis
1
PC
ND
1
PC
GATGGC
mutant(Gly
→
Asp)
2
PC
ND
2
CP
GTTGGC
mutant(Gly
→
Val)
mutant(Gly
Asp)
→
3
PC
GATGGC
3
CP
not-tested
wild type
4
AP
GGTGGC
4
CP
GGTGGC
 wild type
mutant(Gly
Asp)
→
5
PC
GATGGC
5
CP
GGTGGC
 wild type
mutant(Gly
Asp)
→
6
PC
GATGGC
6
IPMN
GATGGC
mutant(Gly
→
Asp)
7
PC
not-tested
 wild type
7
CP
GGTGGC
8
PC
not-tested
8
CP
not-tested
9
PC
not-tested
9
CP
not-tested
mutant(Gly
Asp)
→
10
PC
GATGGC
10
CP
not-tested
mutant(Gly
Asp)
→
11
PC
GATGGC
11
CP
not-tested
12
AP
not-tested
12
CP
not-tested
13
PC
not-tested
13
IPMN
GATGGC
mutant(Gly
→
Asp)
14
CP
ND
14
IPMN
GGTGGC
mutant(Gly
→
Asp)
15
CP
ND
15
IPMN
not-tested
16
CP
ND
16
IPMN
not-tested
17
PET
GGTGGC
wild type
PC, pancreatic cancer; CP, chronic pancreatitis;
AP, autoimmune pancreatitis;
PET, pancreatic endocrine tumor;
IPMN, intraductal papillary mucinous neoplasm;
ND, the DNA sample was not amplified by PCR.
